# Effects of vitamin D replacement combined with Graves’ disease therapy: a retrospective cohort study

**DOI:** 10.55730/1300-0144.5946

**Published:** 2024-10-08

**Authors:** Beyza TAŞKENT SEZGİN, Muhammed KIZILGÜL, Özgür ÖZÇELİK, Taner DEMİRCİ, Hayri BOSTAN, Ümran GÜL, Bekir UÇAN

**Affiliations:** 1 Department of Internal Medicine, Ermenek State Hospital, Karaman, Turkiye; 2 Department of Endocrinology and Metabolism, Ankara Etlik City Hospital, Ankara, Turkiye; 3 Department of Endocrinology and Metabolism, Sakarya University Research and Education Hospital, Sakarya, Turkiye; 4 Department of Endocrinology and Metabolism, Çanakkale Mehmet Akif Ersoy State Hospital, Çanakkale, Turkiye

**Keywords:** Graves’ disease, TSH receptor antibody, vitamin D replacement

## Abstract

**Background/aim:**

In Graves’ disease (GD), an autoimmune disease, antibodies targeting the thyroid stimulating hormone (TSH) receptor cause the production of excessive amounts of thyroid hormone. A significant association was reported between low 25-hydroxy [25(OH)] vitamin D_3_ (VitD) levels and various autoimmune disorders. Therefore, this study aimed to investigate the effects of VitD deficiency and replacement therapy on laboratory and clinical parameters in GD patients.

**Materials and methods:**

Forty GD patients and 37 healthy controls were included in this study. The GD patients were divided into two groups: the nonreplacement group was administered antithyroid treatment only (n = 18), and the replacement group was administered antithyroid treatment + VitD replacement (n = 22). Clinical and laboratory data of all the participants were compared at the time of diagnosis and 3 months after treatment.

**Results:**

Baseline serum VitD levels in the GD patients were significantly lower than the baseline serum VitD levels in the control group (16.1 ± 9.9 vs. 22.2 ± 8.5 ng/mL, p < 0.005). A significant improvement was observed in the serum VitD levels in the replacement group after three months (14.6 ± 8.3 vs. 40.4 ± 17.2 ng/mL, p < 0.001). A significant increase in the serum TSH levels and a significant decrease in the serum free triiodothyronine (fT3) and free thyroxine (fT4) levels were observed in the replacement and nonreplacement groups at the end of three months. However, there was no significant effect of VitD replacement on the serum TSH, fT3, and fT4 levels. There was no difference in the serum thyroid receptor antibodies levels between the replacement and nonreplacement groups.

**Conclusion:**

Although VitD deficiency was detected in the GD patients, there was no significant accelerating effect of VitD replacement on the thyroid hormone levels. These results need to be confirmed with studies that have larger patient numbers and longer follow-up periods.

## 1. Introduction

As an autoimmune disorder, Graves’ disease (GD) causes excessive thyroid hormone production and release by binding antibodies to the thyroid stimulating hormone (TSH), receptor. A multifaceted mechanism, including decreased immunological tolerance to the thyroid, genetic predisposition, and environmental and endogenous variables, contributes to the etiology of the disease [1]. In iodine-sufficient populations, the most widespread cause of overt hyperthyroidism is GD (up to 80%). GD affects 20–30 out of 100,000 individuals each year. Although GD is most common in women between the ages of 30–50 years, it can be diagnosed at any age. If left untreated, GD causes thromboembolic events, reduced bone mineral density, weight loss, and atrial fibrillation [2–4].

In addition to its role in bone and mineral homeostasis, 25**-**hydroxy [25(OH)] vitamin D_3_ (VitD) has significant immunomodulatory effects on the innate as well as adaptive immune systems [5]. It modulates the immune system by limiting the development of proinflammatory cells and controlling the generation of inflammation mediating cytokines. VitD affects immune-related diseases and disorders such as type 1 diabetes, rheumatoid arthritis, psoriasis, multiple sclerosis, systemic sclerosis, systemic lupus erythematosus, and inflammatory bowel diseases [6–8]. Additionally, low serum VitD levels have been shown in neurocognitive dysfunction, cardiovascular diseases, prostate cancer, breast cancer, colon cancer, and thyroid cancer [9–11].

Herein, it was aimed to reveal the effect of VitD replacement in GD patients who began antithyroid treatment on changes in thyroid function and the difference between these patients and those without VitD replacement.

## 2. Materials and methods

### 2.1. Patient groups

GD patients who were followed-up at the Endocrinology and Metabolic Diseases Polyclinic between January 1st, 2021, and June 1st, 2022, were evaluated after confirmation from the Ethics Committee of the Health Sciences University Ankara Dışkapı Yıldırım Beyazıt Training and Research Hospital (123/06 on November 1, 2021). Patients aged 18–65 years who were newly diagnosed with GD or restarted drug treatment due to recurrence and received a weekly dose of 40,000–60,000 IU of VitD were included in the study. Excluded from the study were those who were pregnant, those using medications that could affect VitD levels, those with chronic liver or kidney failure, those with bone metabolism diseases such as primary hyperparathyroidism, rickets, osteoporosis or osteopenia, those using drugs related to bone metabolism and those whose VitD levels were not available at baseline or follow-up. The GD patients were divided into two groups: the nonreplacement group (administered antithyroid treatment only), and the replacement group (administered antithyroid treatment + VitD replacement). Clinical, laboratory, anthropometric, and radiological data of the patients were taken from the patient files and evaluated.

### 2.2. Laboratory

The study included patients who had recently been diagnosed with GD or had experienced a recurrence of the condition. The serum VitD, TSH, free triiodothyronine (fT3), free thyroxine (fT4), antithyroid peroxidase (anti-TPO), antithyroglobuline (anti-TG), thyroid receptor antibodies (TRAb) levels, as well as calcium, phosphorus, magnesium, parathormone, alkaline phosphatase, and albumin values of the participants following 12 h of fasting were recorded. The levels of thyroid function and thyroid antibodies were determined through a direct chemiluminescence immunoassay. An autoanalyzer (Roche Elecsys Vitamin D total II; Roche Diagnostics, Basel, Basel-Stadt, Switzerland) using the chemiluminescence immunoassay method was utilized to measure the concentration of serum VitD. Based on the laboratory results, the VitD status was classified into three categories: VitD deficiency if the level was ≤20 ng/mL, VitD insufficiency if the level was between 20 and 30 ng/mL, and VitD sufficiency if the level was ≥30 ng/mL [8].

### 2.3. Thyroid ultrasonography

The patients underwent an ultrasonography assessment conducted by an experienced endocrinologist using a Hitachi HI VISION Preirus unit (Hitachi Ltd., Chiyoda-ku, Tokyo, Japan) with a 13 MHz linear array transducer both during diagnosis and at the follow-up visits. The thyroid gland volume was calculated using the ellipsoid formula, which involves multiplying the length (cm), width (cm), thickness (cm), and π/6 [9].

### 2.4. Statistical analysis

IBM SPSS Statistics for Windows 22.0 (IBM Corp., Armonk, NY, USA) was used for the data analysis, with various statistical methods employed to determine the distribution and relationships between the variables. Descriptive statistics were utilized to analyze data with a normal distribution, and chi-squared tests were conducted to evaluate the categorical variables. The paired t test and Wilcoxon ordinal sign test were used to compare continuous numerical variables before and after treatment for the normally and nonnormally distributed data, respectively. The effect of VitD replacement therapy on the serum TSH, fT4, and fT3 levels over time was tested using repeated measures analysis of variance. Relationships among the variables that did not follow a normal distribution were investigated using the Spearman’s test. p < 0.05 was accepted as statistically significant.

## 3. Results

**Figure 1:**
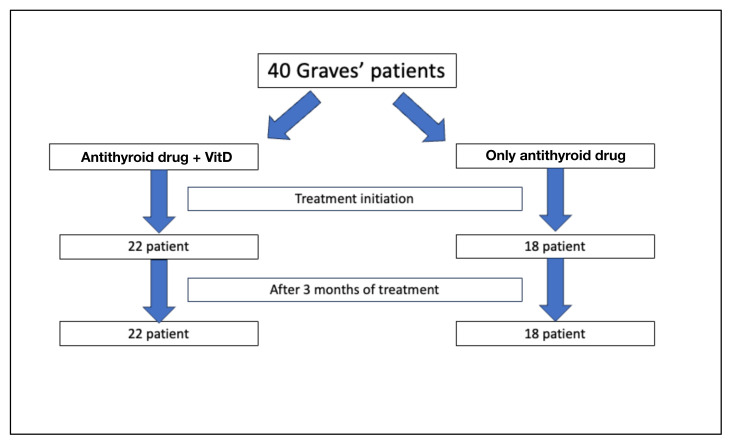
Flow-chart depiction of the study design.

The study included 40 GD patients (11 males, 29 females) with a mean age of 40.2 ± 11.3 years and 37 healthy controls (10 males, 27 females) with a mean age of 33.8 ± 11.1 years (Figure 1). Serum VitD levels were significantly lower in the GD patients compared to the controls (16.1 ± 9.9 to 22.2 ± 8.5 ng/mL, p < 0.005). There were no differences in the age, sex and body mass index between the two groups (Table).

**Figure 2:**
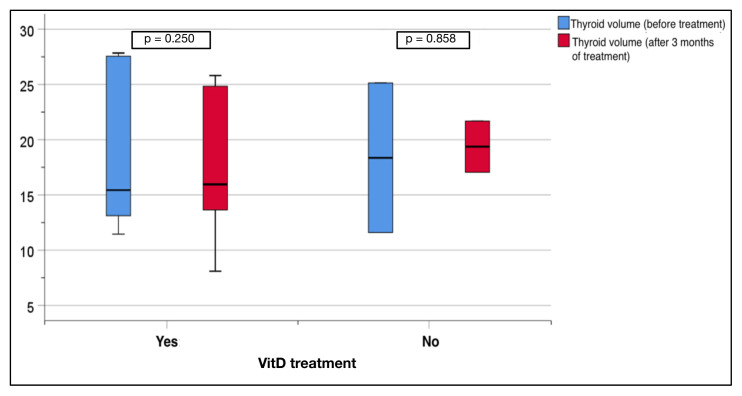
Thyroid volume changes in the replacement and nonreplacement groups.

Three months later, the replacement group demonstrated a notable and statistically significant increase in the VitD levels (14.6 ± 8.3 to 40.4 ± 17.2 ng/mL, p < 0.001), while the nonreplacement group showed no significant change. In the replacement group, there was no significant change in the thyroid volume following treatment (21.5 ± 14.9 mL to 17.4 ± 6.9 mL, p = 0.250). Similarly, in the nonreplacement group, there was no significant change in volume after 3 months of standard treatment (17.7 ± 6.5 to 19.4 ± 3.3 mL, p = 0.858) (Figure 2).

**Figure 3:**
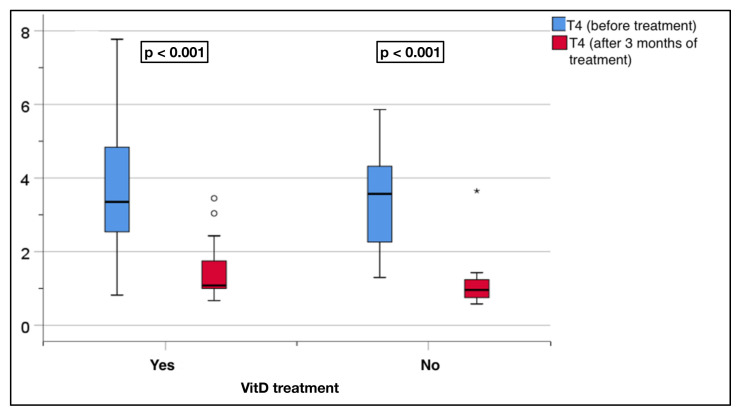
Comparison of the serum TSH levels before and after treatment in the replacement and nonreplacement groups.

In the replacement group, there was a significant increase in the TSH levels after treatment (0.001 mIU/L (0.001–0.020) to 1.015 mIU/L (0.001–6.020), p = 0.002). In the nonreplacement group, there was a significant increase in the TSH levels after treatment (0.001 mIU/L (0.019–0.020) to 2.255 mIU/L (0.001–12,000) p = 0.001) (Figure 3).

**Figure 4:**
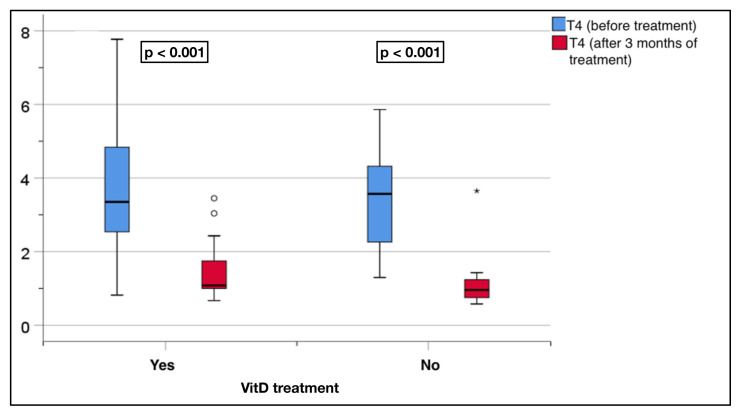
Comparison of the serum fT4 levels before and after treatment in the replacement and nonreplacement groups.

The replacement group showed a significant decrease in the fT4 levels after treatment (3.7 ± 1.8 to 1.4 ± 0.7 pmol/L, p < 0.001). Similarly, the nonreplacement group showed a significant decrease, with a change from 3.4 ± 1.4 to 1.1 ± 0.7 pmol/L (p < 0.001) (Figure 4).

**Figure 5:**
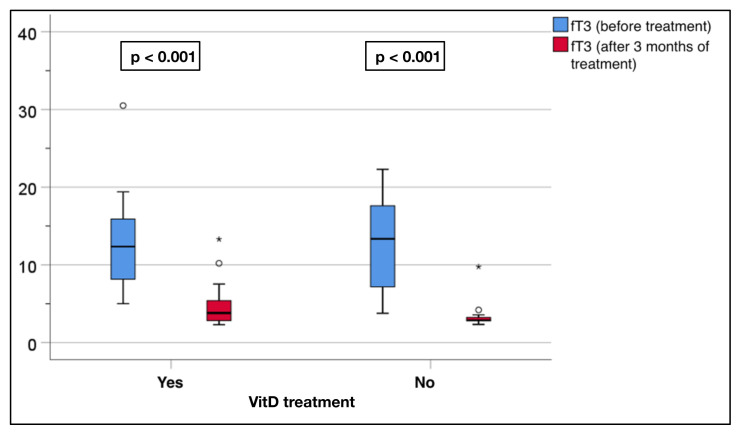
Comparison of the serum fT3 levels before and after treatment in the replacement and nonreplacement groups.

**Figure 6:**
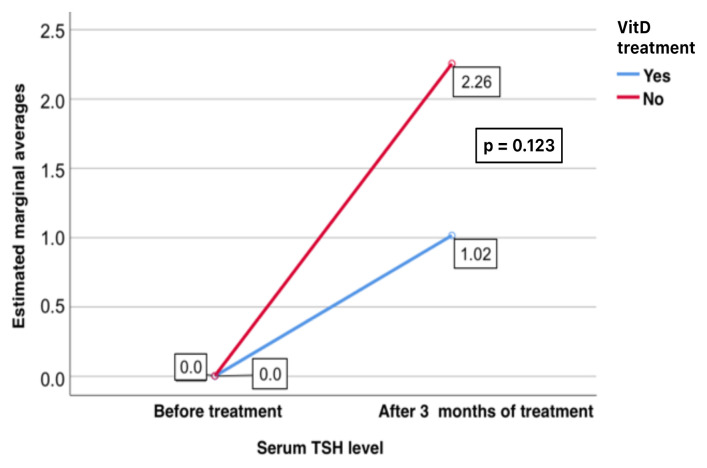
Effect of VitD replacement on changes in the serum TSH levels and the treatment of GD.

**Figure 7:**
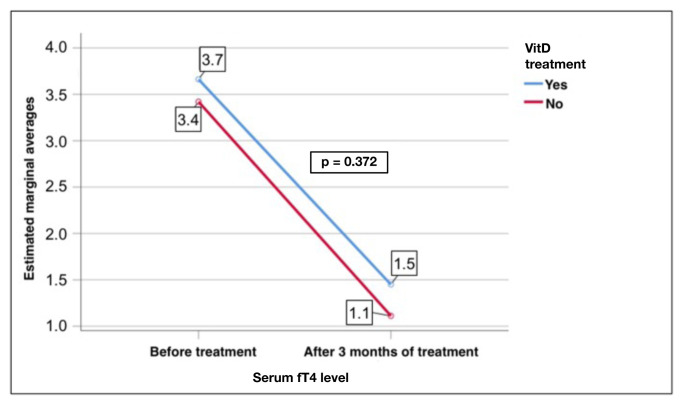
Effect of VitD replacement on changes in the serum fT4 levels and the treatment of GD.

**Figure 8:**
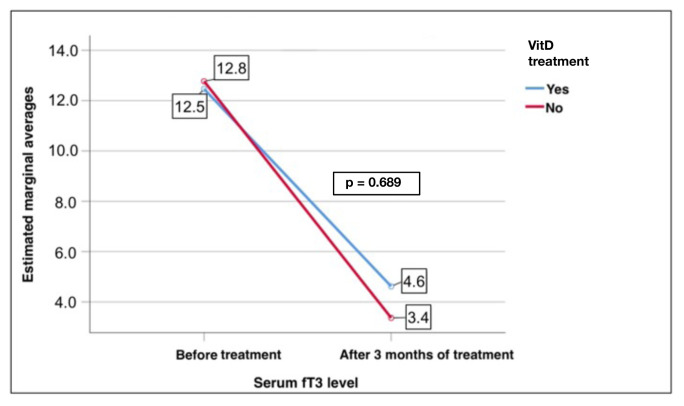
Effect of VitD replacement on changes in the serum fT3 levels and the treatment of GD.

The replacement group also showed a significant decrease in the fT3 levels after treatment (12.5 ± 6.0 to 4.6 ± 2.7 pmol/L, p < 0.001). Similarly, the nonreplacement group showed a significant decrease, with a change from 12.8 ± 6.4 to 3.4 ± 1.7 pmol/L (p < 0.001, Figure 5). However, there was no significant effect of VitD replacement on the TSH, fT4, and fT3 levels in the GD patients (p = 0.23, p = 0.372, and p = 0.689, respectively) (Figures 6–8).

**Figure 9:**
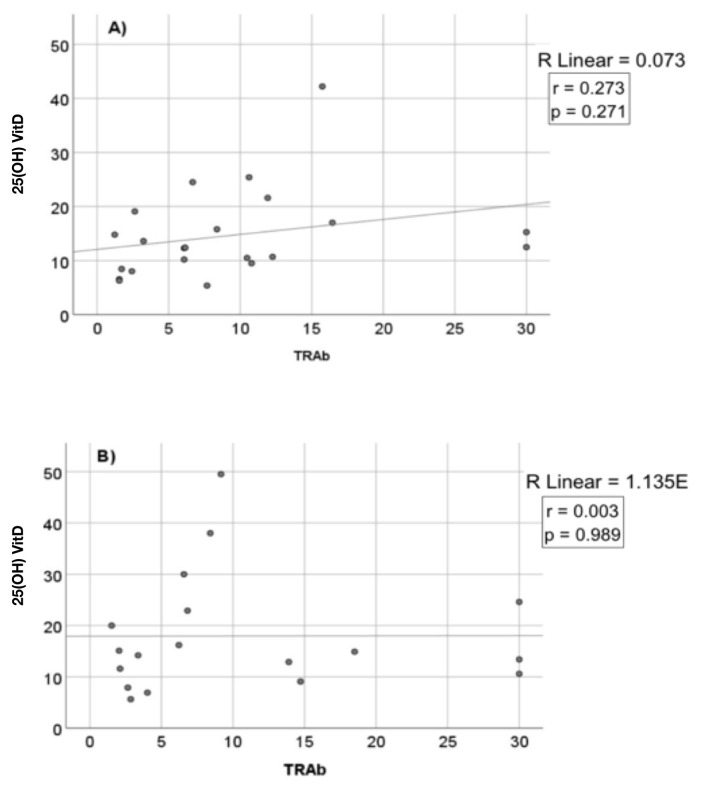
Correlation between the serum TRAb and VitD levels in the replacement (A) and nonreplacement (B) groups before treatment.

**Figure 10:**
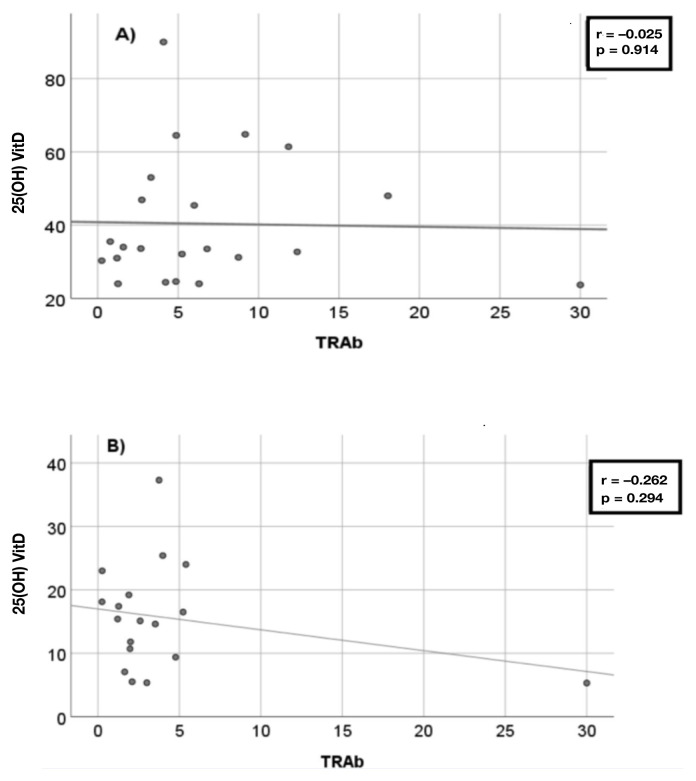
Correlation between the serum TRAb and VitD levels in the replacement (A) and nonreplacement (B) groups after treatment.

Serum TRAb levels between the replacement and nonreplacement groups were similar after 3 months of treatment (6.7 ± 6.8 to 4.2 ± 6.6 IU/L, p = 0.250). Additionally, no significant correlation was observed between the VitD levels and serum TRAb levels before (Figures 9A and 9B) or after (Figures 10A and 10B) treatment in either the replacement (Figures 9A and 10A) or nonreplacement (Figures 9B and 10B) group.

## 4. Discussion

Serum VitD levels of the GD patients were lower than those of the healthy controls. An improvement in the serum TSH, fT3 and fT4 levels was observed in the GD patients who received both antithyroid therapy alone and antithyroid + VitD replacement therapy. However, no significant difference was observed with the VitD replacement therapy alone.

In healthy adults, a strong inverse correlation between TSH and VitD levels has been demonstrated in the euthyroid phase [12]. It was also reported that the incidence of hypovitaminosis and hypocalcemia is high in patients with hypothyroidism. Another study documented that iodine excess is associated with thyroid dysfunction in people with VitD deficiency [13]. Mazokopakis et al. [14] reported an inverse correlation between serum VitD levels and anti-TPO antibody production in patients with hyperthyroidism and normal thyroid function.

Low VitD levels are quite frequently seen in the Turkish population, affecting 93% of individuals [15]. VitD affects immune responses by modulating cytokine production, inhibiting antigen presentation, and promoting immune tolerance. VitD plays a prominent role in regulating the immune system as well as autoimmune diseases, including GD. In addition to studies stating that there is no relationship between VitD levels and GD [16,17], there are studies stating that there is a relationship between them [18–21]. Zhang et al. [22] determined that there was a relationship between VitD levels and TRAb titers. Planck et al. [23] documented that VitD levels were significantly lower in the GD patients compared to the controls, but there was no association between VitD levels and the clinical and laboratory outcomes of GD. Yasuda et al. [19] showed that VitD deficiency was more common in those with GD compared to the controls (32.4% vs. 65%, respectively). Additionally, a significant correlation was found between VitD levels and thyroid volume [24]. Low VitD levels have been associated with a lower likelihood of remission and a higher rate of relapse during treatment with antithyroid medications [25]. In the current study, the serum VitD levels in the GD patients were substantially less than those of the healthy controls. These results were consistent with those of previous studies.

Expanded statistical power and a greater duration of follow-up are required to evaluate the influence of VitD replacement therapies on the management or outcome of GD [26,27]. In a study by Cho and Chung [28], they assessed whether daily VitD replacement could reduce the recurrence of GD. Their research revealed that although recurrence occurred earlier in participants without supplementation, VitD replacement had no appreciable impact on the recurrence frequencies. Grove-Laugesen et al. [29] studied the effect of VitD supplementation in combination with antithyroid therapy on remission in GD patients. They observed that VitD supplementation did not improve the treatment of GD when compared with a placebo in patients with normal and inadequate VitD levels. Behera et al. [30], in their study on patients with hyperthyroidism, administered a dose of 60,000 IU of VitD weekly for eight weeks and then 60,000 IU VitD monthly for four months. At the end of the study, they determined that the VitD and TPO levels increased significantly and the TSH levels decreased significantly. Sheriba et al. [31], in their study evaluating GD patients with VitD deficiency, determined that there was a strong correlation between the thyroid volume, degree of exophthalmos, and VitD levels. It was reported that the administration of 200,000 IU of VitD together with methimazole for three months to DG patients also had positive effects on the reduction of the thyroid volume and degree of exophthalmos. In another recent study, VitD and selenium supplementation in addition to methimazole were administered to control hyperthyroidism in newly diagnosed GD patients with low VitD and selenium levels. It was reported that VitD and selenium supplementation significantly accelerated the control of hyperthyroidism compared to methimazole alone in the GD patients [32]. The effectiveness of VitD replacement therapy alongside antithyroid drug treatment in achieving euthyroidism and reducing thyroid antibody levels was investigated in the current study. However, no significant differences were observed in the thyroid function parameters (TSH, fT4, and fT3) or TRAb levels between the replacement and nonreplacement groups after three months of treatment.

One of the main limitations of this study was its small sample size, which may have hindered the ability to draw robust conclusions and make more generalizable inferences. A larger sample size would allow for more comprehensive statistical analyses and the exploration of potential confounding factors. The second limitation was that the patients were not grouped as those with new diagnosis or recurrence, since only the initiation of antithyroid treatment was considered in the study. It would be useful to compare newly diagnosed or relapsing patients in future studies. The third limitation was that only laboratory and ultrasound evaluations of the patients were performed, and they were not evaluated clinically and the difference between the groups in terms of clinical improvement was determined. The fourth limitation was that the effect of VitD replacement on the drug doses used in the antithyroid therapy was not investigated. Evaluating the effect of VitD replacement on both antithyroid drug doses and clinical improvement will clarify this issue. Another limitation was the short follow-up period. Changes in patients can be distinguished more clearly with longer and more intermittent follow-up to understand the role of VitD, not only in terms of the response to treatment but also in preventing relapse.

In conclusion, while this study confirmed the connection between GD and VitD deficiency and insufficiency, no accelerating effect of VitD supplementation on improvement of the thyroid hormone parameters was observed. A larger-sample and multicenter studies are needed to further investigate the potential benefits of VitD supplementation in GD on meaningful long-term clinical outcomes.

## Figures and Tables

**Table:** Baseline characteristics.

	VitD replacement		p-value
	Yes (n = 22)	No (n = 18)	
Age, year	39.6 ± 10.6	40.8 ± 12.4	0.744
Sex, male, n (%)	5 (22.7)	6 (33.3)	0.347
Body mass index, kg/m^2^	24.6 ± 3.5	25.2 ± 3.5	0.582
Smoking, n (%)	11 (50)	7 (38.9)	0.351
Alcohol, n (%)	1 (4.5)	-	0.550
TSH, (mIU/L), median (min–max)	0.001 (0.001–0.020)	0.001 (0.019–0.020)	0.476
fT4 (pmol/L)	3.7 ± 1.8	3.4 ± 1.4	0.646
fT3 (pmol/L)	12.5 ± 6.0	12.8 ± 6.4	0.877
Thyroid volume, mL	21.5 ± 14.9	17.7 ± 6.5	0.315
TSH receptor antibody (IU/L)	9.3 ± 8.1	10.7 ± 10.1	0.615
25(OH) VitD (ng/mL)	14.6 ± 8.3	18.0 ± 11.5	0.294
